# Prenatal vitamin D and cord blood insulin-like growth factors in Dhaka, Bangladesh

**DOI:** 10.1530/EC-19-0123

**Published:** 2019-05-07

**Authors:** Monika Bilic, Huma Qamar, Akpevwe Onoyovwi, Jill Korsiak, Eszter Papp, Abdullah Al Mahmud, Rosanna Weksberg, Alison D Gernand, Jennifer Harrington, Daniel E Roth

**Affiliations:** 1 Centre for Global Child Health, Hospital for Sick Children, Toronto, Canada; 2 Department of Nutritional Sciences, University of Toronto, Toronto, Canada; 3 Nutrition and Clinical Services Division, icddr,b, Dhaka, Bangladesh; 4 Genetics and Genome Biology, Hospital for Sick Children, Toronto, Canada; 5 Molecular and Medical Genetics, University of Toronto, Toronto, Canada; 6 Department of Paediatrics, Hospital for Sick Children and University of Toronto, Toronto, Canada; 7 Department of Nutritional Sciences, The Pennsylvania State University, University Park, Pennsylvania, USA; 8 Division of Endocrinology, Hospital for Sick Children, Toronto, Canada

**Keywords:** IGF axis, vitamin D, pregnancy, fetal growth

## Abstract

Fetal growth restriction is linked to adverse health outcomes and is prevalent in low- and middle-income countries; however, determinants of fetal growth are still poorly understood. The objectives were to determine the effect of prenatal vitamin D supplementation on the insulin-like growth factor (IGF) axis at birth, to compare the concentrations of IGF-I in newborns in Bangladesh to a European reference population and to estimate the associations between IGF protein concentrations and birth size. In a randomized controlled trial in Dhaka, Bangladesh, pregnant women enrolled at 17–24 weeks of gestation were assigned to weekly oral vitamin D3 supplementation from enrolment to delivery at doses of 4200 IU/week, 16,800 IU/week, 28,000 IU/week or placebo. In this sub-study, 559 woman–infant pairs were included for analysis and cord blood IGF protein concentrations were quantified at birth. There were no significant effects of vitamin D supplementation on cord blood concentrations of IGF-I (*P* = 0.398), IGF-II (*P* = 0.525), binding proteins (BPs) IGFBP-1 (*P* = 0.170), IGFBP-3 (*P* = 0.203) or the molar ratio of IGF-I/IGFBP-3 (*P* = 0.941). In comparison to a European reference population, 6% of girls and 23% of boys had IGF-I concentrations below the 2.5th percentile of the reference population. IGF-I, IGF-II, IGFBP-3 and the IGF-I/IGFBP-3 ratio were positively associated with at least one anthropometric parameter, whereas IGFBP-1 was negatively associated with birth anthropometry. In conclusion, prenatal vitamin D supplementation does not alter or enhance fetal IGF pathways.

## Introduction

Fetal growth restriction (FGR) has been linked to various negative health outcomes, including increased infectious disease susceptibility and increased infant mortality (1). Although the prevalence of FGR worldwide is decreasing, it remains common in many low- and middle-income countries (LMICs), including Bangladesh (2). The high prevalence of FGR necessitates an understanding of the regulation of growth pathways *in utero* in order to develop feasible interventions to improve growth-related outcomes.

The insulin-like growth factor (IGF) axis includes several signal and binding proteins, many of which are under the regulation of growth hormone. IGF-I is the primary regulator of fetal growth, and its circulating concentration is positively associated with size at birth (3, 4, 5, 6). IGF-I exerts its function through binding the IGF-I receptor (IGF-IR) which is expressed throughout human cell lineages, including placental cell types and functions to increase cellular proliferation, differentiation, migration and survival (7). IGF-II is structurally similar to IGF-I and promotes embryonic growth and early development, but it is not believed to play an active regulatory role in postnatal growth under physiological conditions (8). However, IGF-II overexpression leads to postnatal overgrowth, as occurs in Beckwith–Wiedemann syndrome, a disorder characterized by epigenetic overexpression of paternally expressed *IGF2* gene (9). The vast majority (90%) of serum IGFs exist bound to one of six binding proteins that together regulate IGF function (10). The primary binding protein for IGF-I during fetal development is IGF-binding protein 1 (IGFBP-1), which inhibits IGF-I function via its sequestration in circulation (3, 5). Cord blood serum IGFBP-1 concentrations have been found to be inversely associated with size at birth (3, 11). The major IGF-binding protein from infancy into adulthood is IGFBP-3, which is directly related to birth weight, height-for-age z-scores and weight-for-age Z-scores throughout childhood (12, 13, 14, 15). The mechanism of this association is due to increased stability and prolonged half-life of the IGF proteins in the plasma (16).

Variations in IGF axis biomarker concentrations result from a variety of potential factors, such as heritable polymorphisms and ethnicity (17, 18). In pregnancy, insufficient maternal micronutrient intake (particularly iron and calcium) has been associated with reduced fetal IGF-I concentrations (11, 19). Recently, vitamin D has been proposed as a candidate modifiable regulator of the IGF axis. Studies of healthy adult subjects have found positive correlations between serum IGF-I and 25-hydroxyvitamin D (25(OH)D), the major circulating metabolite of vitamin D (20, 21). In a study of children with nutritional rickets, vitamin D supplementation increased serum IGF-I and IGFBP-3 and resulted in accelerated linear growth, suggesting that the treatment of poor growth associated with rickets might be mediated through activation of the IGF axis (13, 22). *In vitro*, vitamin D has been shown to upregulate IGF-I production in mesenchymal stem cells, stimulating osteoblastogenesis (23). A proposed mechanism for vitamin D function in relation to the IGF axis suggests that binding to the vitamin D receptor (VDR) and steroid receptor co-activator (SRC-3) increases IGFBP-3 synthesis in the liver, thereby reducing the clearance of IGF-I and amplifying its function (24). During pregnancy, 25(OH)D is believed to freely cross the placenta unlike the primary active metabolite, 1,25-dihydroxyvitamin D (1,25(OH)2D) (25, 26). The placental and fetal hepatic conversion of 25(OH)D into physiologically active 1,25(OH)2D may have downstream effects on the IGF axis in the fetus and possibly fetal growth (26). In a recent study of pregnant pigs, maternal vitamin D supplementation significantly increased mRNA expression of IGF-II in the muscle tissue of newborn piglets compared to unsupplemented controls and increased prenatal and postnatal skeletal muscle development of the piglets (27). The same study also found that IGF-I expression was decreased in muscle of piglets whose mothers were vitamin D-supplemented when compared to control, contradicting other evidence that suggests a positive correlation between IGF-I and vitamin D levels (27).

To our knowledge, there have been no prior studies of the causal effects of the modulation of prenatal vitamin D status on the fetal IGF-I axis. Leveraging a randomized controlled trial of vitamin D supplementation in pregnancy, the objective of this study was to determine the effect of prenatal vitamin D supplementation on the concentrations of IGF axis proteins (IGF-I, IGF-II, IGFBP-1, IGFBP-3 and the IGF-I/IGFBP-3 molar ratio) at birth in neonates in Bangladesh, where there is a high prevalence of vitamin D deficiency and FGR (2). Secondary objectives were the determination of associations between the IGF biomarkers and birth anthropometry and comparisons of IGF-I concentrations in the study population to reference populations in higher resource settings.

## Materials and methods

This was a sub-study nested within a double-blinded randomized placebo-controlled trial, the Maternal Vitamin D for Infant Growth (MDIG) Trial (Clinical trial registration number: NCT01924013) (28). Written, informed consent was obtained from all women for participation in the MDIG trial, as well as for the storage and use of maternal and infant data, including biological specimens. Detailed methods and primary results of the trial are described elsewhere (28, 29). This study was approved by the Research Ethics Committees at the Hospital for Sick Children in Toronto and the International Centre for Diarrhoeal Disease Research, Bangladesh (icddr,b).

### Subjects

Pregnant women were enrolled at the Maternal and Child Health Training Institute (MCHTI), a government health facility in Dhaka, Bangladesh, between March 2014 and August 2015. Inclusion criteria were pregnant women aged 18 years or older who planned to reside in the study area for at least 18 months, at 17–24 weeks gestation at the time of enrolment (determined either by recalled last menstrual period or by ultrasonography assessment). Exclusion criteria included history of medical conditions or medications that could predispose to vitamin D sensitivity, altered vitamin D metabolism or hypercalcemia. Such medical conditions were self-reported and included tuberculosis, sarcoidosis, renal or ureteral stones, parathyroid disease, renal or liver failure, hypertension, proteinuria and use of antiseizure medication. Women were also excluded if they had a high-risk pregnancy or were unwilling or unable to stop taking non-trial vitamin D or calcium supplements. For the present sub-study, we included mother–infant pairs for whom sufficient venous cord blood samples were available for analysis of all biomarkers (*n* = 559).

### Weekly vitamin D intervention

In the MDIG trial, there were five treatment arms with varying maternal prenatal and postpartum doses of oral vitamin D_3_ supplementation: (1) prenatal placebo followed by placebo post-partum; (2) 4200 IU/week prenatally, followed by placebo postpartum; (3) 16,800 IU/week, followed by placebo postpartum; (4) 28,000 IU/week, followed by placebo postpartum and (5) 28,000 IU/week, up until 6 months postpartum. For the present sub-study, trial arms 4 and 5 were combined due to the identical prenatal doses. Placebo tablets were identical in appearance to those containing vitamin D. All participants were given a co-intervention of 500 mg/day calcium, 66 mg/day elemental iron and 350 µg/day folic acid.

### Specimen and data collection

Within 30 min of delivery of the placenta, blood was collected from the umbilical cord vein by trained study personnel, processed and placed into a portable ultracold freezer (Shuttle ULT-25N; Stirling Ultracold, Athens, OH, USA) and transported to iccdr,b where samples were stored at −70°C. In batches, samples were shipped on dry ice to The Hospital for Sick Children (Toronto, Canada) where they were stored at −80°C. Samples were collected between September 2014 and February 2016 and were maintained at −80°C until batched analysis in 2017.

Birth anthropometry was performed independently by two trained study personnel in duplicate according to rigorous standardized techniques (28); data were eligible to be included in the present analyses if newborn measurements were obtained within 48 h of delivery. Weight was measured to the nearest 5 g using a digital scale. Length was measured using either a Harpenden Infantometer (*n* = 139 infants) or ShorrBoard (*n* = 420). Head circumference was measured using a flexible tape measure. Both head circumference and length were measured to the nearest completed 1 mm. Length-for-age Z-scores (LAZ), weight-for-age Z-scores (WAZ) and head-circumference-for-age Z-scores (HCAZ) were derived using INTERGROWTH-21st (International Fetal and Newborn Growth Consortium for the 21st Century) project standards (30).

### Laboratory methods

IGF-I and IGF-II were measured on samples that were frozen in the field and maintained as such until thawed for analysis. IGFBP-1 and IGFBP-3 analysis was performed on samples that had undergone one previous freeze-thaw cycle. Assessment of the freeze-thaw stability of the binding proteins revealed an average loss of 31% protein concentration from the first to the second freeze-thaw cycle; therefore, absolute concentrations of the binding proteins may be lower than would be observed in samples that had not been previously thawed.

IGF-I and IGF-II were quantified using Quantikine ELISA (DG100 and DG200, R&D Systems), with inter-assay coefficients of variation (CVs) of 17 and 16%, respectively. Intra-assay CVs were 5% for IGF-I and 9% for IGF-II, respectively. IGFBP-1 and IGFBP-3 were quantified simultaneously using a Milliplex magnetic bead panel (HIGFBMAG-53K, Merck KGaA in Darmstadt, Germany, operating as MilliporeSigma in North America). Inter-assay and intra-assay CVs were 11 and 6%, respectively, for IGFBP-1. For IGFBP-3, the inter-assay and intra-assay CVs were 10 and 4%, respectively. All samples beyond the lower and upper limits of detection (LOD) were re-assayed, with the second measurement taken as the true value if it was within the LOD. There were six values that after re-test remained greater than the upper LOD, so the upper LOD was imputed. After re-test, there were no values below the lower LOD.

Since IGF-I cannot bind its receptor when in complex with IGFBP-3, we used the molar ratio of IGF-I to IGFBP-3 to represent the free, and therefore, bioactive IGF-I, as an index of IGF function (16, 31). A criticism of this approach is that it does not take into account the other binding proteins, which may have a significant effect on IGF-I availability and function. However, since the majority of IGF-I is bound to IGFBP-3 in circulation (with the other binding proteins as comparatively minor contributors), this molar ratio can be used as a fairly robust estimate of free IGF-I, and thereby IGF-I function (6).

Maternal concentrations of 25(OH)D at enrolment were ascertained using high-performance liquid chromatography-tandem mass spectrometry, as previously described (28). Average inter-assay and intra-assay CVs were 10 and 5%, respectively.

### Statistical analysis

The study had 80% power to detect a difference of 0.14 standard deviation units in IGF protein concentrations between groups. IGFBP-1, IGFBP-3 and the molar ratio of IGF-I/IGFBP-3 were positively skewed, and thus, were logarithmically transformed before analysis. To estimate the effect of vitamin D on each IGF analyte, ANOVA was used to determine whether there were any differences across groups. This was followed by pairwise analysis using *t*-tests, and the Holm test to adjust for multiple comparisons. Results were expressed as the mean values in each group with 95% CIs. For variables that were logarithmically transformed, geometric mean values were presented, with 95% confidence intervals. We used linear regression models to estimate the associations between each IGF analyte and each anthropometric outcome. Multivariable models adjusted for variables determined a priori by generating a directed acyclic graph; variables included infant sex and gestational age at birth, and maternal factors including age, parity, vitamin D supplementation group, socioeconomic status (asset index, based on item ownership) and body mass index (BMI).

IGF-I concentrations were compared to a reference range based on cord blood samples from a German population (*N* = 15,014) (18). From this sample, the 2.5th percentile was used as the lower limit of the reference range (27 ng/mL for males and 17.9 ng/mL for females) and 97.5th percentile was used as the upper limit (157 ng/mL for males and 125.6 ng/mL for females). This represents 95% of the original population, and it is expected that only 2.5% of the healthy population would fall beyond each of the lower and upper bounds.

The primary analysis used an intent-to-treat approach, including all samples regardless of vitamin D adherence. Previous evidence suggested that IGF-I and IGFBP-3 were associated with gestational age at birth, and as such, a sensitivity analysis was performed to exclude all infants born preterm (<37 weeks, 6% of sample) (32, 33). A per-protocol sensitivity analysis was also performed in which we excluded women that consumed less than 90% of scheduled supplement doses and women that consumed non-study vitamin D or calcium during the prenatal period. Sensitivity analyses excluding samples that were flagged and re-assayed, due to concentrations above or below the assay LOD, were also performed. Subgroup analyses were performed to determine whether effects differed by infant sex or by maternal baseline vitamin D status. All analysis was performed using *Stata* statistical software (StataCorp, 2015). Significance levels of *α* = 0.05 were used throughout.

## Results

In total, 559 woman–infant pairs were included in the study. Baseline characteristics did not differ across supplementation groups (Table 1). The main findings including adverse event rates were previously described (28).

**Table 1:** Maternal and infant baseline characteristics.

Variable	Placebo (*n* = 112)	4200 IU/week (*n* = 108)	16,800 IU/week (*n* = 124)	28,000 IU/week (*n* = 215)	Overall (*n* = 559)
Maternal characteristics					
Age (years), median (IQR)	22 (20–26)	22 (20–25)	22 (19–25)	22 (20–26)	22 (20–26)
BMI (kg/m^2^)^a^, mean (s.d.)	23.7 (4.2)	23.2 (3.8)	23.6 (4.1)	23.6 (3.7)	23.6 (3.9)
Parity, median (IQR)^b^	1 (0–1)	0 (0–1)	1 (0–1)	1 (0–1)	1 (0–1)
Baseline 25(OH)D (nmol/L), mean (s.d.)^c^	26.5 (13.2)	25.1 (13.1)	28.2 (14.1)	27.3 (15.2)	26.9 (14.2)
Infant characteristics					
Gestational age at birth (weeks), median (IQR)	39.1 (38.3–40.0)	39.1 (38.2–40.1)	39.1 (38.1–40.0)	39.4 (38.3–40.3)	39.1 (38.3–40.1)
Preterm (<37 weeks), *n* (%)	5 (5)	7 (7)	11 (9)	11 (5)	34 (6)
Female, *n* (%)	56 (50)	51 (47)	67 (53)	103 (48)	277 (50)

^a^BMI was measured mid-pregnancy, and thus, is only used for the purposes of between-group comparisons within the study. ^b^Parity represents the number of viable pregnancies completed before the index pregnancy. ^c^N_placebo_ = 110; N_4200 IU/week_ = 107; N_16,800 IU/week_ = 123; N_28,000 IU/week_ = 211; N_overall_ = 551.

### Effect of vitamin D supplementation on IGF concentrations

There were no significant differences across treatment groups in venous cord plasma IGF-I, IGF-II, IGFBP-1 and IGFBP-3 concentrations or in the molar ratio of IGF-I/IGFBP-3 (Table 2). However, among infants born to mothers who were vitamin D deficient at baseline (defined as serum 25(OH)D <30 nmol/L), 4200 IU/week and 28,000 IU/week led to significantly greater IGFBP-1 concentrations compared to placebo (Supplementary Table 1, see section on supplementary data given at the end of this article). Also, the 28,000 IU/week group had significantly greater IGFBP-3 concentrations when compared to placebo (*P* = 0.006); however, this finding was not robust against the removal of two extreme IGFBP-3 outliers (Supplementary Table 2). None of the remaining subgroup or sensitivity analyses demonstrated significant effects of vitamin D on cord IGF biomarker concentrations (Supplementary Tables 3, 4, 5 and 6).

**Table 2:** Insulin-like growth factor (IGF) axis protein concentrations in cord plasma, by supplementation group.

Protein	Placebo	4200 IU/week	16,800 IU/week	28,000 IU/week	*P* value^a^
IGF-I					
*N*	112	108	124	215	
Mean (95% CI), ng/mL	43.2 (39.1, 47.4)	40.3 (36.6, 44.0)	43.7 (40.3, 47.1)	44.2 (41.6, 46.8)	0.398
IGF-II					
*N*	111	107	124	214	
Mean (95% CI), ng/mL	436 (398, 474)	396 (359, 432)	422 (380, 464)	420 (394, 446)	0.525
IGFBP-1^b^					
*N*	109	107	123	210	
Geometric mean (95% CI), ng/mL	32.6 (26.6, 40.0)	43.7 (34.1, 56.1)	33.6 (27.2, 41.5)	40.0 (34.2, 46.8)	0.17
IGFBP-3^b^					
*N*	107	105	122	209	
Geometric mean (95% CI), ng/mL	444 (398, 497)	424 (387, 464)	462 (425, 503)	480 (448, 516)	0.203
IGF-I/IGFBP-3 molar ratio^b,c^					
*N*	107	105	122	209	
Geometric mean (95% CI), ng/mL	32.8 (28.5, 37.7)	31.3 (27.4, 35.8)	31.9 (28.4, 35.8)	31.2 (28.3, 34.4)	0.941

^a^*P* values are for global tests of differences across treatment groups, using ANOVA. ^b^Analyses were conducted for IGFBP-1, IGFBP-3 and IGF-I/IGFBP-3 ratio after logarithmically transforming biomarkers. ^c^Molar ratio = (IGF-I (nmol/L))/(IGFBP-3 (nmol/L)) × 100, where IGF-I (nmol/L) = IGF-I (ng/mL) × 0.1307 and IGFBP-3 (nmol/L) = IGFBP-3 (ng/mL) × 0.03478.

### Associations between IGF proteins and birth size

IGF-I was positively associated with LAZ, WAZ and HCAZ, and IGF-II was positively associated with LAZ and WAZ (Table 3). IGFBP-1 was negatively associated with LAZ, WAZ and HCAZ; however, the association with LAZ was not significant in the multivariable model. IGFBP-3 was positively associated with LAZ. The molar ratio of IGF-I to IGFBP-3 was positively associated with WAZ and HCAZ. Overall, IGF-I, IGF-II, IGFBP-3 and the IGF-I/IGFBP-3 molar ratio were positively associated with at least one anthropometric parameter, whereas IGFBP-1 was negatively associated with all three anthropometric parameters (Table 3).

**Table 3:** Associations between anthropometric parameters at birth and IGF axis biomarker concentrations.

Biomarker	*N*	Unadjusted models		Multivariable adjusted models^a^	
		Effect estimate^b^ (95% CI)	*P* value	Effect estimate^b^ (95% CI)	*P* value
IGF-I (ng/mL)					
LAZ	492	0.132 (0.090, 0.174)	<0.001	0.117 (0.074, 0.161)	<0.001
WAZ	496	0.193 (0.159, 0.226)	<0.001	0.169 (0.135, 0.202)	<0.001
HCAZ	497	0.119 (0.078, 0.159)	<0.001	0.099 (0.058, 0.140)	<0.001
IGF-II (ng/mL)					
LAZ	490	0.056 (0.012, 0.100)	0.012	0.053 (0.010, 0.096)	0.018
WAZ	494	0.045 (0.008, 0.083)	0.017	0.041 (0.006, 0.076)	0.023
HCAZ	495	0.007 (−0.035, 0.049)	0.731	0.002 (−0.039, 0.043)	0.923
IGFBP-1^c^					
LAZ	482	−0.009 (−0.016, −0.001)	0.023	−0.006 (−0.013, 0.002)	0.149
WAZ	486	−0.017 (−0.023, −0.011)	<0.001	−0.012 (−0.018, −0.006)	<0.001
HCAZ	487	−0.012 (−0.019, −0.005)	<0.001	−0.010 (−0.017, −0.003)	0.006
IGFBP-3^c^					
LAZ	481	0.029 (0.013, 0.045)	<0.001	0.029 (0.013, 0.044)	<0.001
WAZ	485	0.013 (−0.001, 0.027)	0.071	0.012 (−0.001, 0.024)	0.081
HCAZ	486	0.007 (−0.008, 0.023)	0.354	0.006 (−0.009, 0.021)	0.406
IGF-I/IGFBP-3 molar ratio^c^					
LAZ	481	0.008 (−0.004, 0.021)	0.173	0.005 (−0.008, 0.017)	0.467
WAZ	485	0.033 (0.023, 0.043)	<0.001	0.027 (0.017, 0.037)	<0.001
HCAZ	486	0.020 (0.009, 0.032)	0.001	0.016 (0.005, 0.027)	0.005

^a^Multivariable model adjusted for sex, gestational age at birth, asset index, maternal age, maternal BMI, parity, vitamin D supplementation group. ^b^Effect estimate represents the average change in anthropometric outcome for every 10 ng/mL change in IGF-I, for every 100 ng/mL change in IGF-II, and for every 10% change in concentration of IGFBP-1, IGFBP-3 and the molar ratio of IGF-I/IGFBP-3. ^c^IGFBP-1, IGFBP-3 and the molar ratio of IGF-I/IGFBP-3 were logarithmically transformed before analysis. LAZ, length-for-age Z-scores; WAZ, weight-for-age Z-scores; HCAZ, head circumference-for-age z-scores.

### Cord blood IGF-I concentrations in Bangladesh compared to an external reference

**Figure 1:**
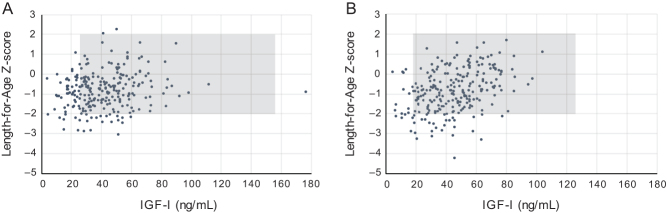
Cord blood IGF-I concentrations and newborn length-for-age z-scores (LAZ) for boys (A) and girls (B) in Dhaka, Bangladesh (*n* = 245 for boys; *n* = 247 for girls). Reference ranges for IGF-I and LAZ are shaded in gray, from the 2.5th to 97.5th percentiles of the reference population for IGF-I and −2 to +2 z-scores for LAZ. For IGF-I, the reference range was based on Bidlingmaier *et al*. (18), using 27–157 ng/mL for males and 17.9–125.6 ng/mL for females. LAZ was based on the INTERGROWTH-21st neonatal size standards; the range shown is from −2 to 2 z-score.

In the present study population, mean IGF-I values were 41.7 ng/mL for boys and 44.6 ng/mL for girls (*P* = 0.087 for comparison between sexes). In our initial protocol, only placebo group values were planned to be compared to the reference range; however, since there was no difference in IGF protein concentrations in response to vitamin D supplementation, the entire cohort was compared to the reference interval. As such, 23% of boys in the study were below the 2.5th percentile lower bound of the IGF-I sex-specific reference interval, where 6% of girls were below the sex-specific interval. Only one boy was above the upper limit of the IGF-I reference interval, and no girls were above their respective upper limit. Figure 1 shows an approximation of the expected distribution of IGF-I and LAZ in a reference population and the empirical distribution in the present study population, demonstrating that the Bangladesh cohort was shifted to both lower LAZ and IGF-I values.

## Discussion

We did not find an overall effect of prenatal vitamin D supplementation on cord blood concentrations of IGF proteins in a population with a high prevalence of vitamin D deficiency. In the primary analysis of the MDIG trial, vitamin D supplementation did not have any significant effect on fetal or infant growth; however, we had speculated that anthropometric parameters may not be sufficiently responsive to changes in micronutrient status and that vitamin D deficiency may have subclinical or biochemical effects on growth-related pathways in the absence of effects on infant length or weight (29, 34). However, this hypothesis was not supported, as the lack of effects of vitamin D on fetal IGF markers were consistent with the overall null effects of the MDIG trial on infant size. We conclude that there is a lack of evidence to support the notion that maternal prenatal vitamin D supplementation enhances activation of the IGF axis in the fetus.

Given previous associations between vitamin D and the IGF axis in observational studies in children and adults, it is unclear why there was no effect of prenatal supplementation on cord blood concentrations of IGF markers (13, 20, 21, 22). It is possible that other factors (e.g., systemic inflammation) masked an effect or that the effects of vitamin D deficiency were compensated for by another factor (for example, the co-supplemented calcium) (35). In a subgroup analysis, we were surprised to find that vitamin D supplementation had a positive effect on IGFBP-1 concentrations in infants who were born to mothers who were vitamin D deficient at baseline. Since IGFBP-1 is negatively correlated with size at birth, this finding would suggest that vitamin D supplementation could have a negative effect on fetal growth in women with prenatal vitamin D deficiency. However, the subgroup effects should be viewed cautiously, particularly since there was no clear dose dependency. This may have been a chance finding or alternatively there could be compensatory endocrine pathways that overcome subtle negative effects of vitamin D on IGF-I availability.

The confirmation of previously reported IGF–anthropometric associations in the present study establishes confidence in the robustness of the measured IGF concentrations (12). A difference of 10 ng/mL (approximately 0.5 standard deviation units) in IGF-I was associated with an average increase in LAZ of 0.13. Although this is a modest effect size, this association represents a plausible causal mechanism to partly explain between-individual variability in fetal growth within this population. However, we noted that the entire distribution of IGF-I values was shifted down in this population, which corresponded to the negatively shifted LAZ distribution relative to the INTERGROWTH-21 standard. This suggests that the causes of low IGF-I, and thus, FGR in this population were widespread in the community, rather than limited to a subset of vulnerable infants, consistent with the notion of growth faltering in LMICs as a whole-population condition (36). There is no evidence to suggest that IGF-I concentrations are strongly determined by ethnicity; for example, Patel *et al*. found that British-born South Asian newborns had IGF-I values that were well above those we observed in Bangladesh and were more consistent with the published German reference range: 60.9 ng/mL for boys and 86.4 ng/mL for girls, compared to the medians in the present study of 39.8 ng/mL for boys and 44.5 ng/mL for girls (14). That study also did not find a significant difference in IGF-I values between British-born South Asian children and White European children.

A higher percentage of boys in the present study were considered to have low IGF-I values compared to girls, suggesting that boys may be more sensitive than girls to the factors suppressing IGF-I and growth in this population. Although our study did not find significant differences between male and female cord blood IGF-I concentrations, a previous study of Indian newborns revealed that girls had significantly higher IGF-I at birth than boys (51.4 ng/mL compared to 42.9 ng/mL; reported *P* < 0.03) (37).

The strengths of this study include the randomized controlled dose-ranging design of the trial, the measurement of multiple IGF axis markers, the rigorous measurement of newborn anthropometry (28) and the confirmation of previously established correlations between IGF and birth anthropometry. One limitation of this study was the lack of an international IGF-I standard for external comparison of observed values. Yet, there are no validated, widely accepted reference ranges for IGF-I values in cord blood; furthermore, variations in concentration between studies may result from the use of different assays. There is a lack of established reference ranges for the other IGF proteins, thus, we were unable to compare these proteins to benchmark concentrations and could only perform within-study comparisons. A second limitation was the analysis of binding proteins on samples which had been previously thawed. Despite the overall loss of protein, there was high within-assay precision and we assumed that any effect of degradation would be similar across treatment groups and would not affect our inferences. Further, in our study sample, the proteins still held their expected associations with anthropometric outcomes, increasing our confidence in the use of these values. A third limitation was that supplementation in the MDIG trial began in the second trimester, yet, evidence of placental regulation of vitamin D metabolism has led to the hypothesis that effects of vitamin D on fetal growth may depend on preconceptional or early gestational supplementation, which should be further explored in future studies (38).

In conclusion, maternal prenatal vitamin D supplementation did not affect the fetal IGF axis. Based on the results of this study, there is no evidence to support supplementation with vitamin D starting mid-pregnancy would alter these growth pathways *in utero*. It is unknown if vitamin D starting earlier in pregnancy or prepregnancy would impact fetal growth. Further work should identify modifiable factors responsible for the shift of IGF to lower values in this population, as well as the cause for the apparent greater vulnerability of boys to growth-stunting stressors.

## Supplementary Material

Supplemental Table 1: Insulin-like growth factor (IGF) axis protein concentrations in cord plasma by supplementation group, stratified by maternal vitamin D status at baseline (25(OH)D<30nmol/L vs. ≥30nmol/L).

Supplemental Table 2: Insulin-like growth factor binding protein 3 (IGFBP-3) protein concentration in cord plasma1 by supplementation group, in the subset of mothers with deficient vitamin D status as baseline (defined as 25(OH)D<30nmol/L), before and after the exclusion of 2 influential outliers.

Supplemental Table 3: Insulin-like growth factor (IGF) axis protein concentrations in cord plasma by supplementation group, stratified by infant sex.

Supplemental Table 4: Insulin-like growth factor (IGF) axis protein concentrations in cord plasma by supplementation group, excluding all infants born preterm.

Supplemental Table 5: Insulin-like growth factor (IGF) axis protein concentrations in cord plasma by supplementation group, excluding all samples that were flagged for re-assaying*.

Supplemental Table 6: Per protocol analysis depicting insulin-like growth factor (IGF) axis protein concentrations in cord plasma, by supplementation group.

## Declaration of interest

The authors declare that there is no conflict of interest that could be perceived as prejudicing the impartiality of the research reported.

## Funding

The MDIG trial was funded by the Bill and Melinda Gates Foundation (OPP1066764).
