# Supplementary material for: Prenatal vitamin D and cord blood insulin-like growth factors in Dhaka, Bangladesh
Source: Endocr Connect. 2019 May 7;8(6):745–53. doi: 10.1530/EC-19-0123 (PMC6547305; doi:10.1530/EC-19-0123)
Supplement: Supplemental Table 1: Insulin-like growth factor (IGF) axis protein concentrations in cord plasma by supplementation group, stratified by maternal vitamin D status at baseline (25(OH)D<30nmol/L vs. ≥30nmol/L). [file supplementary_table_1.pdf]

**Supplemental Table 1**: Insulin-like growth factor (IGF) axis protein concentrations in cord plasma by supplementation group, stratified by maternal vitamin D status at baseline (25(OH)D<30nmol/L vs. ≥30nmol/L).

| Protein | Placebo | | 4,200 IU/week | | 16,800 IU/week | | 28,000 IU/week | | Overall p-value[1] |
|---|---|---|---|---|---|---|---|---|---|
| | N | Mean (95% CI) | N | Mean (95% CI) | N | Mean (95% CI) | **N** | Mean (95% CI) | |
| **IGF-I, ng/mL** | | | | | | | | | |
| 25(OH)D<30 nmol/L | 70 | 41.9 (37.6,46.2) | 72 | 37.5 (32.8,42.2) | 77 | 43.7 (39.1,48.2) | 141 | 42.8 (39.7,45.9) | 0.191 |
| 25(OH)D≥30 nmol/L | 40 | 45.2 (36.3,54.1) | 35 | 46.1 (40.3,51.9) | 46 | 44.4 (39.3,49.6) | 70 | 46.4 (41.6,51.2) | 0.961 |
| **IGF-II, ng/mL** | | | | | | | | | |
| 25(OH)D<30 nmol/L | 70 | 445 (392,497) | 71 | 390 (347,432) | 77 | 430 (373,486) | 140 | 433 (400,465) | 0.410 |
| 25(OH)D≥30 nmol/L | 39 | 428 (373,482) | 35 | 401 (329,473) | 46 | 410 (347,474) | 70 | 398 (350,447) | 0.900 |
| **IGFBP-1, ng/mL[2]** | | | | | | | | | |
| 25(OH)D<30 nmol/L | 68 | 27.6 (21.9,34.8) | 71 | 53.4 (38.6,73.8) [a] | 76 | 40.0 (30.0,53.4) | 137 | 44.5 (36.7,53.9) [a] | 0.009 |
| 25(OH)D≥30 nmol/L | 39 | 45.1 (30.4,67.0) | 35 | 27.8 (19.5,39.7) | 46 | 25.7 (19.1,34.6) | 69 | 33.8 (25.4,45.1) | 0.114 |
| **IGFBP-3, ng/mL[2]** | | | | | | | | | |
| 25(OH)D<30 nmol/L | 68 | 414 (355,481) | 70 | 444 (397,496) | 75 | 475 (422,534) | 135 | 505 (470,543) [a] | 0.042 |
| 25(OH)D≥30 nmol/L | 37 | 524 (450,610) | 34 | 381 (323,449) | 46 | 445 (394,503) | 70 | 451 (386,527) | 0.100 |
| **IGF-I/IGFBP-3 molar ratio[2, †]** | | | | | | | | | |
| 25(OH)D<30 nmol/L | 68 | 34.4 (28.2,41.8) | 70 | 26.9 (22.6,32.0) | 75 | 30.8 (26.4,35.9) | 135 | 28.8 (25.9,32.1) | 0.185 |
| 25(OH)D≥30 nmol/L | 37 | 28.7 (24.1,34.1) | 34 | 43.4 (36.8,51.3) [a] | 46 | 34.6 (29.0,41.4) | 70 | 34.7 (28.5,42.1) | 0.075 |

[1] Global p-value for differences across treatment groups, using ANOVA.
[2] Analyses were conducted for IGFBP-1, IGFBP-3, and IGF-I/IGFBP-3 ratio after logarithmically transforming biomarkers. Geometric means with 95% confidence intervals are shown.
[a] Post-hoc pairwise comparisons using t-tests showed significant pairwise difference from group receiving placebo, after adjusting for multiple comparisons using the Holm test.
**†** Molar ratio = (IGF-I(nmol/L))/(IGFBP-3 (nmol/L))×100, where IGF-I(nmol/L) = IGF-I (ng/mL)×0.1307 and IGFBP-3(nmol/L)=IGFBP-3(ng/mL)×0.03478
