# Supplementary material for: Prenatal vitamin D and cord blood insulin-like growth factors in Dhaka, Bangladesh
Source: Endocr Connect. 2019 May 7;8(6):745–53. doi: 10.1530/EC-19-0123 (PMC6547305; doi:10.1530/EC-19-0123)
Supplement: Supplemental Table 2: Insulin-like growth factor binding protein 3 (IGFBP-3) protein concentration in cord plasma1 by supplementation group, in the subset of mothers with deficient vitamin D status as baseline (defined as 25(OH)D<30nmol/L), before and after the exclusion of 2 influential outliers. [file supplementary_table_2.pdf]

Supplemental Table 2: Insulin-like growth factor binding protein 3 (IGFBP-3) protein concentration in cord plasma[1] by supplementation group, in the subset of mothers with deficient vitamin D status as baseline (defined as 25(OH)D<30nmol/L), before and after the exclusion of 2 influential outliers.

Protein (n)	Placebo	4,200 IU/week	16,800 IU/week	28,000 IU/week	Overall p-value[2]
**Before Exclusion of Influential Observations**					
N	68	70	75	135	-
Geometric mean (95%CI), ng/mL	414 (355,481)	444 (397,496)	475 (422,534)	505 (470,543) [a]	0.042
**After Exclusion of Influential Observations**					
N	66	70	75	135	-
Geometric mean (95%CI), ng/mL	448 (404,497)	444 (397,496)	475 (422,534)	505 (470,543)	0.163

[1] Analyses were conducted for IGFBP-3 after logarithmic transformation.
[2] Global p-value for differences across treatment groups, using ANOVA.
[a] Post-hoc pairwise comparisons using t-tests showed significant pairwise difference from group receiving placebo, after adjusting for multiple comparisons using the Holm test.
