# Supplementary material for: Prenatal vitamin D and cord blood insulin-like growth factors in Dhaka, Bangladesh
Source: Endocr Connect. 2019 May 7;8(6):745–53. doi: 10.1530/EC-19-0123 (PMC6547305; doi:10.1530/EC-19-0123)
Supplement: Supplemental Table 3: Insulin-like growth factor (IGF) axis protein concentrations in cord plasma by supplementation group, stratified by infant sex. [file supplementary_table_3.pdf]

Supplemental Table 3: Insulin-like growth factor (IGF) axis protein concentrations in cord plasma by supplementation group, stratified by infant sex.

Protein	Placebo		4,200 IU/week		16,800 IU/week		28,000 IU/week		Overall p-value[1]
	N	Mean (95% CI)	N	Mean (95% CI)	N	Mean (95% CI)	**N**	Mean (95% CI)	
**IGF-I, ng/mL[2]**									
Boys	55	43.2 (36.4,50.0)	57	39.3 (34.3,44.2)	57	38.6 (34.0,43.1)	112	43.8 (40.1,47.5)	0.301
Girls	56	43.3 (38.3,48.3)	51	41.4 (35.8,47.0)	67	48.0 (43.2,52.9)	103	44.6 (41.1,48.2)	0.282
**IGF-II, ng/mL[2]**									
Boys	54	450 (383,516)	56	406 (346,467)	57	451 (388,513)	112	413 (374,452)	0.554
Girls	56	426 (384,467)	51	384 (344,422)	67	398 (341,454)	102	428 (392,464)	0.441
**IGFBP-1, ng/mL[a]**									
Boys	54	36.8 (27.9,48.6)	56	59.6 (42.0,84.5)	57	45.4 (33.8,61.1)	109	41.8 (33.2,52.6)	0.164
Girls	54	28.9 (21.2,39.6)	51	31.1 (22.1,44.0)	66	25.9 (19.3,34.6)	101	38.2 (30.8,47.4)	0.173
**IGFBP-3, ng/mL[a]**									
Boys	54	449 (383,526)	55	436 (390,488)	56	474 (419,536)	108	468 (430,509)	0.758
Girls	52	443 (376,522)	50	410 (353,477)	66	453 (402,510)	101	494 (440,556)	0.255
**IGF-I/IGFBP-3 molar ratio[a,†]**									
Boys	54	32.1 (26.1,39.4)	55	29.5 (25.0,34.9)	56	28.0 (23.5,33.3)	108	31.7 (28.2,35.6)	0.607
Girls	52	33.2 (27.2,40.6)	50	33.5 (27.0,41.5)	66	35.7 (30.6,41.6)	101	30.7 (26.2,36.0)	0.634

[1] Global p-value for differences across treatment groups, using ANOVA.
[2] Means are arithmetic means with 95% confidence intervals
[a] Analyses were conducted for IGFBP-1, IGFBP-3, and IGF-I/IGFBP-3 ratio after logarithmically-transforming biomarkers. Geometric means with 95% confidence intervals are shown.
[†] Molar ratio = (IGF-I(nmol/L))/(IGFBP-3 (nmol/L))×100, where IGF-I(nmol/L) = IGF-I (ng/mL)×0.1307 and IGFBP-3(nmol/L)=IGFBP-3(ng/mL)×0.03478
