# Supplementary material for: Prenatal vitamin D and cord blood insulin-like growth factors in Dhaka, Bangladesh
Source: Endocr Connect. 2019 May 7;8(6):745–53. doi: 10.1530/EC-19-0123 (PMC6547305; doi:10.1530/EC-19-0123)
Supplement: Supplemental Table 4: Insulin-like growth factor (IGF) axis protein concentrations in cord plasma by supplementation group, excluding all infants born preterm. [file supplementary_table_4.pdf]

Supplemental Table 4: Insulin-like growth factor (IGF) axis protein concentrations in cord plasma by supplementation group, excluding all infants born preterm.

Protein	Placebo	4,200 IU/week	16,800 IU/week	28,000 IU/week	Overall p-value[1]
**IGF-I** [2]					
N	107	101	113	204	
Mean (95% CI), ng/mL	43.7 (39.4,48.0)	40.4 (36.5,44.2)	43.9 (40.2,47.5)	44.1 (41.4,46.7)	0.456
**IGF-II** [2]					
N	106	100	113	203	
Mean (95% CI), ng/mL	444 (405,483)	395 (357,433)	424 (379,469)	414 (389,440)	0.376
**IGFBP-1** [a]					
N	104	101	112	201	
Geometric mean (95% CI), ng/mL	34.1 (27.7,42.0)	45.1 (35.1,58.1)	35.5 (28.3,44.6)	41.2 (35.1,48.3)	0.259
**IGFBP-3** [a]					
N	102	99	111	200	
Geometric mean (95%CI), ng/mL	444 (396,499)	421 (383,462)	459 (419,502)	479 (445,516)	0.221
**IGF-I/IGFBP-3 molar ratio** [a,†]					
N	102	99	111	200	
Geometric mean (95% CI), ng/mL	33.1 (28.6,38.3)	31.4 (27.3,36.0)	32.3 (28.6,36.4)	31.2 (28.2,34.5)	0.902

[1] Global p-value for differences across treatment groups, using ANOVA.

[2] Means are arithmetic means with 95% confidence intervals

[a] Analyses were conducted for IGFBP-1, IGFBP-3, and IGF-I/IGFBP-3 ratio after logarithmically-transforming biomarkers. Geometric means with 95% confidence intervals are shown.

[†] Molar ratio = (IGF-I(nmol/L))/(IGFBP-3 (nmol/L))×100, where IGF-I(nmol/L) = IGF-I (ng/mL)×0.1307 and IGFBP-3(nmol/L)=IGFBP-3(ng/mL)×0.03478
