# Supplementary material for: Prenatal vitamin D and cord blood insulin-like growth factors in Dhaka, Bangladesh
Source: Endocr Connect. 2019 May 7;8(6):745–53. doi: 10.1530/EC-19-0123 (PMC6547305; doi:10.1530/EC-19-0123)
Supplement: Supplemental Table 5: Insulin-like growth factor (IGF) axis protein concentrations in cord plasma by supplementation group, excluding all samples that were flagged for re-assaying*. [file supplementary_table_5.pdf]

Supplemental Table 5: Insulin-like growth factor (IGF) axis protein concentrations in cord plasma by supplementation group, excluding all samples that were flagged for re-assaying[*].

Protein (n)	Placebo	4,200 IU/week	16,800 IU/week	28,000 IU/week	Overall p-value[1]
**IGF-I [2]**					
N	110	107	124	215	
Mean (95% CI), ng/mL	42.1 (38.8,45.5)	40.1 (36.4,43.7)	43.7 (40.3,47.1)	44.2 (41.6,46.8)	0.284
**IGF-II [2]**					
N	110	106	123	214	
Mean (95% CI), ng/mL	434 (396,472)	396 (359,432)	422 (380,464)	420 (394,446)	0.580
**IGFBP-1 [a]**					
N	104	102	121	204	
Geometric mean (95% CI), ng/mL	30.3 (25.2,36.5)	39.7 (31.0,50.8)	32.1 (26.2,39.3)	38.1 (32.6,44.5)	0.187
**IGFBP-3 [a]**					
N	103	102	121	201	
Geometric mean (95%CI), ng/mL	459 (423,498)	418 (381,458)	461 (423,502)	476 (442,512)	0.179
**IGF-I/IGFBP-3 molar ratio [a, †]**					
N	102	101	121	201	
Geometric mean (95% CI), ng/mL	31.6 (28.0,35.6)	31.9 (27.8,36.5)	31.8 (28.3,35.8)	31.8 (28.8,35.1)	0.999

[1] Global p-value for differences across treatment groups, using ANOVA.

[2] Means are arithmetic means with 95% confidence intervals

[a] Analyses were conducted for IGFBP-1, IGFBP-3, and IGF-I/IGFBP-3 ratio after logarithmically-transforming biomarkers. Geometric means with 95% confidence intervals are shown.

[†] Molar ratio = (IGF-I(nmol/L))/(IGFBP-3 (nmol/L))×100, where IGF-I(nmol/L) = IGF-I (ng/mL)×0.1307 and IGFBP-3(nmol/L)=IGFBP-3(ng/mL)×0.03478

[*] Values were flagged if they were beyond the upper or lower limits of detection of their respective assay
