# Supplementary material for: Prenatal vitamin D and cord blood insulin-like growth factors in Dhaka, Bangladesh
Source: Endocr Connect. 2019 May 7;8(6):745–53. doi: 10.1530/EC-19-0123 (PMC6547305; doi:10.1530/EC-19-0123)
Supplement: Supplemental Table 6: Per protocol analysis depicting insulin-like growth factor (IGF) axis protein concentrations in cord plasma, by supplementation group. [file supplementary_table_6.pdf]

Supplemental Table 6: Per protocol analysis depicting insulin-like growth factor (IGF) axis protein concentrations in cord plasma, by supplementation group.

Protein (n)	Placebo	4,200 IU/week	16,800 IU/week	28,000 IU/week	Overall p-value[1]
**IGF-I** [2]					
N	109	97	114	198	
Mean (95% CI), ng/mL	43.7 (39.5,47.8)	40.7 (36.8,44.6)	42.8 (39.2,46.4)	43.5 (40.8,46.2)	0.676
**IGF-II** [2]					
N	108	96	114	197	
Mean (95% CI), ng/mL	439 (400,478)	397 (358,435)	413 (374,452)	420 (392,447)	0.504
**IGFBP-1**[a]					
N	106	96	113	193	
Geometric mean (95% CI), ng/mL	31.9 (26.1,39.2)	40.1 (31.3,51.4)	35.7 (28.7,44.6)	40.3 (34.3,47.4)	0.337
**IGFBP-3** [a]					
N	104	94	112	193	
Geometric mean (95%CI), ng/mL	438 (391,491)	416 (376,460)	473 (434,516)	488 (452,526)	0.066
**IGF-I/IGFBP-3 molar ratio** [a,†]					
N	104	94	112	193	
Geometric mean (95% CI), ng/mL	33.6 (29.2,38.7)	32.6 (28.4,37.4)	30.4 (27.0,34.1)	30.2 (27.3,33.4)	0.538

[1] Global p-value for differences across treatment groups, using ANOVA.

[2] Means are arithmetic means with 95% confidence intervals

[a] Analyses were conducted for IGFBP-1, IGFBP-3, and IGF-I/IGFBP-3 ratio after logarithmically-transforming biomarkers. Geometric means with 95% confidence intervals are shown.

[†] Molar ratio = (IGF-I(nmol/L))/(IGFBP-3 (nmol/L))×100, where IGF-I(nmol/L) = IGF-I (ng/mL)×0.1307 and IGFBP-3(nmol/L)=IGFBP-3(ng/mL)×0.03478
